# Discovery and Genomic Characterisation of Novel Papillomaviruses in Australian Wild Birds

**DOI:** 10.3390/pathogens14060514

**Published:** 2025-05-22

**Authors:** Subir Sarker, Vasilli Kasimov, Md. Mizanur Rahaman, Babu Kanti Nath, Martina Jelocnik

**Affiliations:** 1 Biomedical Sciences & Molecular Biology, College of Medicine and Dentistry, James Cook University, Townsville, QLD 4811, Australia; mdmizanur.rahaman@my.jcu.edu.au; 2 Institute for Biomedicine and Glycomics, Griffith University, Southport, QLD 4215, Australia; 3 Biosecurity Research Program and Training Centre, Gulbali Institute, Charles Sturt University, Wagga Wagga, NSW 2678, Australia; bnath@csu.edu.au; 4 School of Science, Technology and Engineering, University of the Sunshine Coast, Sippy Downs, QLD 4557, Australia; mjelocni@usc.edu.au; 5 Centre for Bioinnovation, University of the Sunshine Coast, Sippy Downs, QLD 4557, Australia

**Keywords:** papillomavirus, genomics, evolution, phylogenetics, wild birds

## Abstract

Papillomaviruses are small, circular DNA viruses that infect epithelial and mucosal cells, which have co-evolved with their hosts over time. While certain mammalian papillomaviruses—especially those linked to disease—are well studied, there is limited knowledge about papillomaviruses associated with avian species. In this study, we identified two avian papillomaviruses from eye/choana swabs of the sacred kingfisher (*Todiramphus sanctus*) and the little corella (*Cacatua sanguinea*), collected in Queensland, Australia. The genomes of these viruses, designated as todiramphus sanctus papillomavirus 1 (TsPV1) and cacatua sanguinea papillomavirus 1 (CsPV1), were found to be 7883 and 7825 base pairs in length, respectively. The TsPV1 and CsPV1 genomes exhibited the highest nucleotide sequence identity (>56%) with papillomavirus genomes previously sequenced from mallards or wild ducks in the United States, followed by those from black-legged kittiwakes and Atlantic puffins (>54%) in Newfoundland, Canada. Both TsPV1 and CsPV1 share approximately a 65% nucleotide sequence identity in the L1 gene with anas platyrhynchos papillomavirus 3 (AplaPV3), indicating that they represent novel avian papillomaviruses. Notably, the two genomes in this study were nearly identical (99.69%), and their L1 proteins shared 100% sequence identity. Phylogenetic analysis positioned TsPV1 and CsPV1 within a clade of avian papillomaviruses associated with closely related avian hosts, including the mallard, African grey parrot, common chaffinch, and Atlantic canary. These findings underscore the importance of further research on studying additional Australian bird species longitudinally, which will help to establish potential disease associations and ecological impacts of previously unrecognised and novel papillomaviruses in Australian wild birds.

## 1. Introduction

The *Papillomaviridae* family comprises a broad and diverse group of non-enveloped, double-stranded DNA viruses that infect a wide variety of vertebrate species, including mammals, birds, reptiles, and fish [1,2,3,4,5,6,7,8]. As classified by the International Committee on Taxonomy of Viruses (ICTV), this family currently consists of more than 50 recognised genera and over 340 species [9,10]. While many papillomavirus (PV) infections are asymptomatic, certain PVs can cause benign epithelial growths, such as warts, which can sometimes develop into malignant lesions, including squamous cell carcinoma and other cancers affecting the skin or mucosa [2,3,4,5,6]. Although human papillomaviruses (HPVs) have been the most thoroughly studied within the *Papillomaviridae* family, advances in genomic sequencing have identified various PV types affecting other animal species, including birds. Avian papillomaviruses (APVs) represent a distinct and important subgroup within the *Papillomaviridae* family. They are currently classified within the genera *Thetapapillomavirus*, *Etapapillomavirus*, *Dyoepsilonpapillomavirus*, *Dyozetapapillomavirus*, *Treisepsilonpapillomavirus*, and *Treiszetapapillomavirus*. Despite their taxonomic recognition, APVs remain comparatively underexplored. Infected birds generally develop benign papillomas on their skin or mucosa, with the impact on their health varying according to the size and location of the lesions.

Structurally, APVs share similarities with other PVs, featuring a double-stranded, circular DNA genome typically between 5.7 and 8.6 kilobases (kb), enclosed within an icosahedral capsid [10]. The genome encodes several open reading frames (ORFs) that encode both structural proteins (L1 and L2) and non-structural proteins (E1–E9) involved in viral replication, cellular transformation, and capsid assembly [11,12]. The PV genome also contains a regulatory segment known as the long control region (LCR), with some viruses having an additional non-coding region. Although PVs share a conserved genomic structure, diversity in the E proteins exists across species, while the essential ORFs—E1, E2, L2, and L1—are consistently present across all PV genomes [13]. Despite these genetic similarities, APVs differ in evolutionary history and host specificity, making them valuable for studies in comparative viral genomics.

Traditionally, PVs were considered highly host-specific, evolving in close association with their host species, suggesting a pattern of host-specific codivergence. However, recent studies indicate that cross-species transmission may be more common than previously believed, especially among closely related host species [14,15,16]. For example, cases of cross-species transmission, such as bovine PVs infecting other herbivores and certain felids, suggest that host specificity in PVs may be more flexible than once assumed [17,18].

While much of the research on PV diversity has focused on mammals, there remains limited understanding of APVs. The earliest confirmed APV cases date to the 1970s, with PV-like particles detected in squamous papillomas in chaffinches using electron microscopy [19]. Subsequently, APVs have been found in other bird species, including the African grey parrot and the northern fulmar, in association with cutaneous tumours or lesions [20,21,22]. While the clinical manifestations of APV infections are generally benign, certain strains have shown the capacity for more invasive disease, particularly when co-infections or environmental stressors are present [19,20,21,22]. Recent advances in next-generation sequencing and molecular diagnostics are facilitating the identification, characterisation, and study of APV diversity, shedding light on their evolutionary pathways and potential for cross-species transmission.

This study reports the complete genome sequences and molecular characterization of two novel APVs detected in swabs collected from the sacred kingfisher (*Todiramphus sanctus*) and the little corella (*Cacatua sanguinea*). These findings improve our understanding of APV diversity, host–virus interactions, and evolutionary dynamics, which are essential for understanding broader implications for avian health and disease ecology.

## 2. Materials and Methods

### 2.1. Sampling and DNA Extraction

A subset of DNA samples (*n* = 11) from four wild avian groups—parrots, pigeons, kingfishers, and raptors—was selected for further investigation (see Supplementary Table S1 for details). Notably, the majority of these DNA samples harboured multiple avian pathogens, including *Chlamydiaceae*, beak and feather disease virus (BFDV), avipoxviruses, columbid alphaherpesvirus 1 (CoAHV1), and psittacid alphaherpesvirus 1 (PsAHV1) (Supplementary Table S1) [23]. These birds were admitted to the Australia Zoo Wildlife Hospital (AZWH, Beerwah, QLD, Australia) for various health issues, including clinical diseases and trauma. The attending veterinarians managed the pre-sampling, admission, care, and euthanasia processes. Approval for sampling from the euthanized birds was granted by the University of the Sunshine Coast Animal Research Ethics Committee (ANE1940, ANE2057) [23]. Genomic DNA was extracted from swab samples collected from the eye, liver, or eye/choana (Supplementary Table S1) using the QiaAMP DNA Mini Kit, following the manufacturer’s guidelines (Qiagen, Clayton, VIC, Australia). The eluted DNA was stored at −20 °C for subsequent analysis [23].

### 2.2. Next-Generation Sequencing

The quantity and quality of the extracted DNA were assessed using a Qubit dsDNA high-sensitivity assay kit with Qubit Fluorometer v4.0 (Thermo Fisher Scientific, Waltham, MA, USA). Library construction was performed using the Illumina DNA Prep (Illumina, San Diego, CA, USA) as per kit instructions, starting with 200 ng of DNA quantified using the Qubit Fluorometer v4.0 (Thermo Fisher Scientific, USA). The quality and quantity of the prepared library were evaluated by the Australian Genome Research Facility, Melbourne, Australia. Cluster generation and the sequencing of the library were performed with 150 bp paired-end reads on the Illumina^®^ NovaSeq chemistry, according to the manufacturer’s instructions.

### 2.3. Bioinformatic Analyses

The resulting raw sequencing reads were analysed as per the established pipeline [24,25] using Geneious Prime^®^ (version 2023.1.1, Biomatters, Auckland, New Zealand). Briefly, preliminary quality evaluation for all raw reads was generated and pre-processed to remove ambiguous base calls and poor-quality reads, and trimmed to remove the Illumina adapter sequences. Trimmed sequence reads were mapped against the chicken genome *Gallus gallus* (GenBank accession no. NC006088.5) to remove likely host DNA contamination. In addition, the reads were further mapped to the *Escherichia coli* bacterial genomic sequence (GenBank accession no. U00096) to remove possible bacterial contamination. The remaining cleaned and unmapped reads were used for de novo assembly using a SPAdes assembler (version 3.10.1) [26] in Geneious. The resulting contigs were compared against the nonredundant nucleotide and protein databases on GenBank using BLASTN and BLASTX [27], respectively, with an E-value threshold of 1 × 10^−5^ to remove potential false positives. Contigs that had significant BLAST (version 2.16.0) hits with bacteria, eukaryotes, or fungi were filtered out to remove non-viral reads. Virus contigs of interest greater than 500 nucleotides (nt) were imported into Geneious Prime^®^ (version 2023.1.1) for further functional analysis. The detected viruses were annotated using Geneious Prime^®^ (version 2023.1.1), where genus-specific published viruses were used as a reference guideline.

### 2.4. Comparative Genomics and Phylogenetic Analysis

The genomic comparisons of the newly sequenced complete viral genomes of papillomaviruses were visualised using clinker [28], Base-by-Base [29], and Geneious (version 2023.1.1). The pairwise sequence similarities between the selected PVs sequences were identified against representative avian papillomaviruses sequences by Base-by-Base and MAFFT software (Version 11.0.11) [29,30,31].

Phylogenetic analysis of the novel PV genome sequences identified in this study was conducted alongside selected papillomavirus genome sequences available in the GenBank database. APV sequences were downloaded from GenBank in July 2024 (Table 1). The amino acid sequences of the L1 gene and the nucleotide sequences of the selected complete PVs genomes were aligned using MAFFT (version 7.450) with the G-INS-i algorithm (gap open penalty 1.53; offset value 0.123) within Geneious. Maximum likelihood (ML) phylogenetic trees were constructed using LG (L1 gene) and GTR (complete genome) substitution models with 1000 bootstrap replicates in Geneious. Human papillomavirus 41 (GenBank accession number, X56147) was used as an outgroup.

## 3. Results

### 3.1. Genomes of Two Novel Avian Papillomaviruses

Two complete papillomavirus (PV) genomes were detected in DNA extracted from the eye tissue of a sacred kingfisher (*T. sanctus*) and a little corella (*C. sanguinea*), corresponding to a prevalence rate of 18.18%. The samples were collected from separate locations in Queensland—Peachester (sacred kingfisher) in October 2020 and Burpengary (little corella) in November 2020. The genomes measured 7883 bp (average coverage of 260.68×) and 7825 bp (average coverage of 10.20×) in length, respectively. The GC content for the todiramphus sanctus papillomavirus 1 (TsPV1) and cacatua sanguinea papillomavirus 1 (CsPV1) complete genome is 57.8%. The genomes of TsPV1 and CsPV1 sequenced in this study showed the highest nucleotide sequence identity (>56%) with a PV genome sequenced from a mallard in the United States (GenBank accession no. PP057987) [7], followed by a black-legged Kittiwake (>54%) from Newfoundland Canada (GenBank accession no. MK620305) [6] and an Atlantic puffin from Newfoundland Canada (>54%) (GenBank accession no. MK620302) (Table 1). In addition, the two PV genomes sequenced in this study were almost identical (99.69% nucleotide identity at the genomic level).

### 3.2. Comparative Analyses

**Figure 1:**
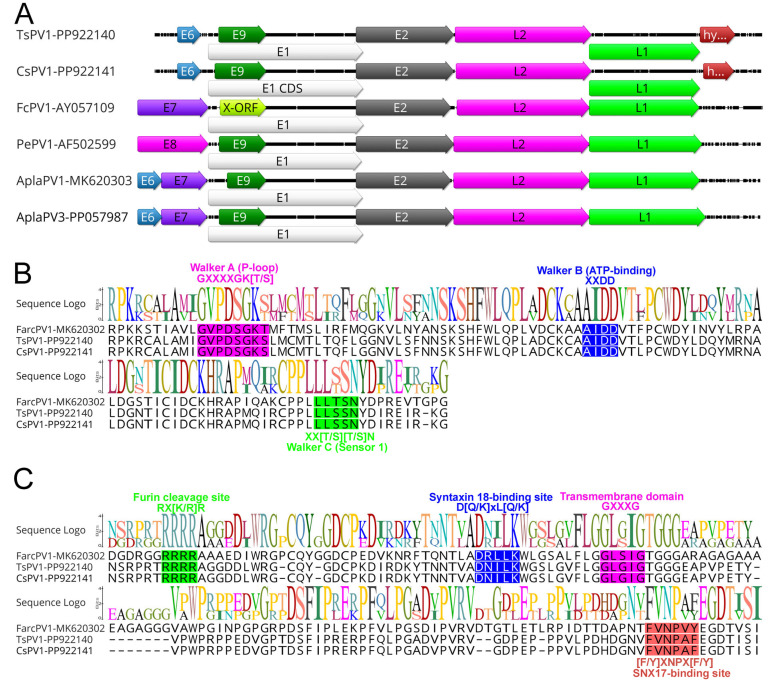
(**A**) A genomic illustration of TsPV1 and CsPV1 in comparison with the selected APVs, visualized using Geneious Prime. The arrows depict the direction of the transcription of genes and open reading frames (ORFs). Each ORF of the APV genomes is colour coded and homologous genes are shown using their specific colour. Hyp indicates genes encoding a hypothetical protein. The Walker domains of the E1 helicase are highlighted in panel (**B**) (alignment positions 530–640), while specific protein domains of the L2 protein are shown in panel (**C**) (alignment positions 1–190). The domain designation and typical sequences of each motif are indicated below and above the alignments. TsPV1 and CsPV1 are the novel types identified in this study.

Like other papillomaviruses, APVs identified in this study exhibited the characteristic genome structure, including the four core ORFs encoding the proteins L1, L2, E1, and E2. The ORFs for the E6 and E9 proteins, commonly found in most papillomaviruses, were also present. Additionally, an extra ORF encoding the hypothetical protein was identified (Figure 1A and Table 2).

The hexameric DNA helicase E1, the only enzyme encoded by papillomaviruses, is also the most conserved protein across these viruses [32]. In APVs, the E1 proteins range from 587 to 722 amino acids in length, slightly larger than those found in mammalian papillomaviruses, which typically span 600–650 amino acids [6]. Similarly to other papillomaviruses, the E1 proteins of TsPV1 and CsPV1 were 702 amino acids long, sharing the highest sequence identity with duck papillomavirus 3 (64.84%, GenBank accession no. QBR99468.1). Notably, the E1 proteins of TsPV1 and CsPV1 are identical, showing 100% amino acid identity. The E1 gene in both TsPV1 and CsPV1 encodes a typical bipartite nuclear localisation signal within the N-terminal region (sequence: _187_RSKNSMPKRNAAGAIQVHGHDAAAPKRVRGP_217_), which has a predicted score of 6.1 as determined by cNLS mapper [33]. As illustrated in Figure 1B, the E1 gene also encodes several conserved motifs common to the AAA+ (ATPases associated with diverse cellular activities) protein family, including Walker A (phosphate-binding loop: GXXXXGK[T/S]; specifically, **G**VPDS**GKS**), Walker B (ATP-binding domain: XXDD; represented as AI**DD**), and Walker C (sensor 1: XX[T/S][T/S]N; represented as XX**SSN**). These motifs—highlighted by their conserved residues (shown in bold)—are well conserved across APV helicase domains.

Additionally, the DNA-binding domain, located within the C-terminal region of the E2 protein, serves as a key regulator of viral transcription and replication [34]. This sequence (GXTXQ[L/V]KTIRXR; position in TsPV1 and CsPV1, _325_GYTGQLKTIRHR_336_) is also highly conserved across all papillomaviruses.

The L2 protein of the TsPV1 and CsPV1 showed the highest identity with duck papillomavirus (>44%, GenBank accession no. ANN29878.1) sequenced from India in 2014 [35]. Distinctive conserved motifs were identified within the L2 structural protein across all APVs (Figure 1C). Among these were the furin cleavage motif (RX[K/R]R) located in the N-terminal region of both TsPV1 and CsPV1, along with a variable number of transmembrane GXXXG domains and a sorting nexin 17 (SNX17)-binding site ([F/Y]XNPX[F/Y]). The furin cleavage site is crucial for viral entry, while the transmembrane domains and SNX17-binding motif are likely involved in endosomal escape [12]. Additionally, a syntaxin 18-binding site (D[Q/K]xL[Q/K]), which facilitates viral transport toward the nucleus [12], was also found in both TsPV1 and CsPV1.

The major capsid protein (L1) of papillomavirus in both TsPV1 and CsPV1 was 1551 bp in nucleotide length, displaying the highest amino acid similarity with duck papillomavirus (>65%, GenBank accession no. QBR99472), which was sequenced in India in 2014 [35]. The L1 proteins of TsPV1 and CsPV1 were found to be 100% identical.

### 3.3. Evolutionary Relationships of APVs

**Figure 2:**
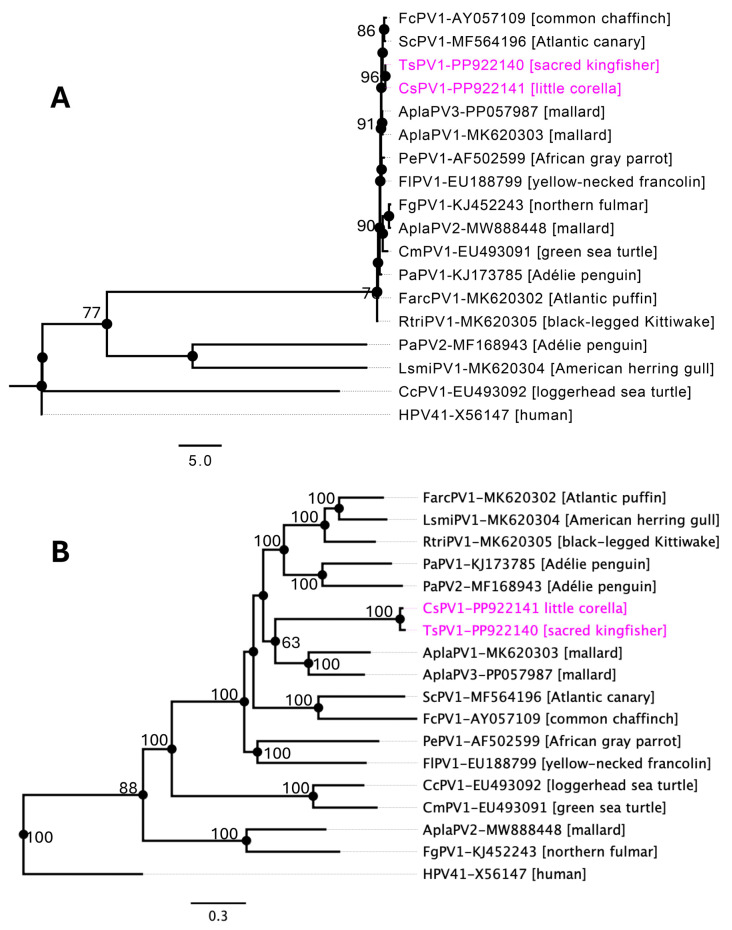
Phylogenetic relationships between papillomaviruses detected in this study and other selected APVs. Maximum likelihood (ML) trees were constructed from multiple alignments of the L1 gene (**A**) and nucleotide sequences of the selected complete PVs genomes (**B**) using Geneious Prime (version 2023.1.1). The numbers on the branches represent bootstrap values as percentages. The labels at the branch tips refer to abbreviated virus names and GenBank accession numbers, followed by the host species in parentheses. The positions of the APVs detected in this study are highlighted in pink. Bootstrap values lower than 60 are not shown. Human papillomavirus 41 (GenBank accession number, X56147) was used as an outgroup. The details of the APVs used in the phylogenetic tree are provided in Table 1.

Phylogenetic analysis, using both the individual L1 gene (Figure 2A) and the complete genome sequences of the selected PVs (Figure 2B), provides support for the classification of the newly identified APVs in this study. The phylogenetic tree based on the individual L1 gene sequences of the selected APVs (Figure 2A) demonstrated a similar topology to the maximum likelihood (ML) tree generated from the complete genome sequences of the selected papillomaviruses (Figure 2B). As shown in Figure 2, both trees supported the formation of a subclade comprising papillomaviruses infecting the mallard (AplaPV1 and AplaPV3), African grey parrot (PePV1), common chaffinch (FcPV1), and Atlantic canary (ScPV1), reflecting their potential phylogenetic relationships. However, the phylogenetic positions of the TsPV1 and CsPV1 detected in this study lacked strong bootstrap support (maximum of 63%), indicating no obvious close relationship with other known papillomaviruses. This suggests that these viruses may represent an intermediate evolutionary lineage distinct from the previously identified avian papillomaviruses.

### 3.4. Taxonomic Identification

According to the International Committee for the Taxonomy of Viruses (ICTV), papillomavirus classification is determined through a combination of nucleotide identity thresholds for the L1 gene and the corresponding phylogenetic analysis. The ICTV has established distinct phylogenetic cut-offs for demarcating taxa—60% for genera, 70% for species, and 90% for types [10]. The average pairwise identities between the L1 nucleotide sequences of each APV type were calculated and are shown in Table 3.

**Figure 3:**
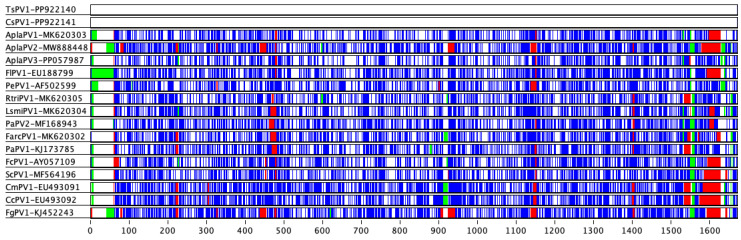
A visual comparison of L1 gene amino acids from the selected avian papillomaviruses using Base-By-Base. Differences and indels in the L1 gene between PV sequences in this study (TsPV1 and CsPV1) and other avian papillomaviruses are shown in different colours (green = insertion, blue = substitution, red = deletion).

The nucleotide sequence identity of the L1 gene of TsPV1 and CsPV1 was 100%, but they were found to be highly divergent compared to other APVs (Table 3 and Figure 3). Specifically, the L1 gene of TsPV1 and CsPV1 shared approximately 64% pairwise identity with AplaPV1 and AplaPV3, and around 60% identity with members of the genus *Etapapillomavirus*, including ScPV1 and FcPV1. \Based on these sequence similarities, TsPV1 and CsPV1 likely belong to the same genus as AplaPV1 and AplaPV3 (which is yet to be assigned). However, the detected papillomaviruses likely meet the criteria to be classified under a novel papillomavirus genus, with the highest pairwise identity being approximately 64%.

## 4. Discussion

This study reports the characterisation of two novel avian papillomaviruses (APVs), TsPV1 and CsPV1, detected from swabs taken from the eye tissues of the sacred kingfisher and little corella, respectively. Both viruses exhibit typical papillomavirus genomic structures, comprising four core open reading frames (ORFs) for L1, L2, E1, and E2, as well as the additional E6 and E9 ORFs found in most papillomaviruses. The presence of conserved motifs and structural elements across both genomes, along with the high nucleotide identity observed between them (99.69%), suggests these viruses are closely related and may represent a unique evolutionary lineage within the avian papillomaviruses.

Although papillomaviruses have been extensively studied in humans and certain domestic animals, their prevalence and ecological dynamics in wild avian species remain relatively underexplored. This study’s finding of a relatively high prevalence rate (18.18%) of papillomaviruses in the sampled avian population is particularly noteworthy. The observed prevalence aligns with earlier reports suggesting that papillomaviruses may be more widespread among avian species than previously recognised [6,13]. Several factors may contribute to these elevated prevalence rates, including the host species’ ecological characteristics, environmental conditions, and the mechanisms of virus transmission within bird populations. For instance, gregarious or communal species such as the sacred kingfisher (*T. sanctus*) and the little corella (*C. sanguinea*) may facilitate viral spread through close physical contact or shared habitats [36].

In terms of genomic sequence identity, TsPV1 and CsPV1 demonstrated the highest similarity to papillomaviruses found in other avian species, with >56% nucleotide identity to a virus isolated from a mallard (wild duck) in the United States [7], and approximately 54% similarity to viruses in black-legged kittiwakes and Atlantic puffins from Canada [6]. The relatively low sequence identities compared to other APVs and their high similarity to each other suggest these viruses may be members of a distinct, previously unrecognized genus within the *Papillomaviridae* family. Moreover, the L1 gene of TsPV1 and CsPV1 shared approximately 64% pairwise identity with AplaPV1 and AplaPV3. Based on these sequence similarities, TsPV1 and CsPV1 are likely members of distinct species within the same genus as AplaPV1 and AplaPV3.

Our analysis highlights the E1 protein as a critical component for both TsPV1 and CsPV1, aligning with its role as the most conserved enzyme in papillomaviruses [32]. The E1 proteins of both viruses are 702 amino acids long, closely matching the sizes of other avian E1 proteins [6,7,37], which are generally longer than those of mammalian papillomaviruses [38]. The presence of conserved motifs, including the Walker A, B, and C domains, indicates that these viruses retain key functional domains essential for viral replication and helicase activity, which is necessary for efficient viral DNA replication.

The DNA-binding domain within the E2 protein, which regulates viral transcription and replication [34], is also highly conserved across TsPV1 and CsPV1, with the sequence _325_GYTGQLKTIRHR_336_ being consistent with known APVs [6,7,37]. These conserved sequences highlight the evolutionary importance of E2’s regulatory function in the viral life cycle, particularly for maintaining control over viral genome replication and transcription [34].

In the L2 protein, conserved motifs such as the furin cleavage site, transmembrane GXXXG domains, and the SNX17- and syntaxin 18-binding motifs are consistent with those found in other APVs, suggesting conserved mechanisms for host cell entry, endosomal escape, and nuclear transport [12]. These findings underscore the critical role of the L2 protein in facilitating multiple steps of the infection process, from entry into host cells to trafficking within the host cytoplasm and nucleus.

Phylogenetic analyses, based on both the L1 gene and complete PVs genome sequences, reveal that TsPV1 and CsPV1 likely occupy an intermediate evolutionary position among APVs. The lack of strong bootstrap support in their phylogenetic positioning reflects their divergence from established avian papillomaviruses, which may indicate that these viruses represent an early branch or a novel sub-lineage. This is further supported by their high pairwise identity to each other and relatively low identity to other APVs, with the highest similarity in the L1 gene at ~64%—significantly below the threshold for species demarcation but close to the genus boundary. Moreover, according to the ICTV’s classification criteria [10], the L1 gene nucleotide divergence of TsPV1 and CsPV1 supports their designation as members of a new papillomavirus genus as AplaPV1, AplaPV3, distinct from currently known avian PV lineages. These findings contribute valuable insights into the diversity and evolutionary history of APVs and highlight the existence of yet-unknown viral lineages in avian species. This study also acknowledges the limitation of not including traditional viral isolation experiments. This constraint primarily stems from the initial limited sampling strategy, which was specifically designed for viral metagenomic analysis rather than for culture-based virus isolation.

## 5. Conclusions

The identification of TsPV1 and CsPV1 enriches our understanding of APVs, particularly those infecting non-traditional avian hosts such as the sacred kingfisher and little corella. The close genetic relationship between TsPV1 and CsPV1, coupled with their unique phylogenetic placement, suggests they may represent a novel papillomavirus genus. Future studies involving a broader range of avian hosts and geographic regions are essential to clarify the diversity and evolutionary pathways of APVs and to further elucidate the functional roles of conserved motifs in viral pathogenesis and host adaptation.

## Figures and Tables

**Table 1:** Comparative genomic analysis of representative APVs.

Papillomavirus Name	Genus	Species	GenBank Accession Numbers	Length (nt)	Host	Abbreviation	Pairwise Genomic Identity (%) _TsPV1	Pairwise Genomic Identity (%) _CsPV1
Todiramphus sanctus papillomavirus 1	*unclassified*	*unclassified*	PP922140.1	7883	*Todiramphus sanctus* (Sacred kingfisher)	TsPV1		99.69
Cacatua sanguinea papillomavirus 1	*unclassified*	*unclassified*	PP922141.1	7825	*Cacatua sanguinea* (Little corella)	CsPV1	99.69	
Psittacus erithacus papillomavirus 1	*Thetapapillomavirus*	*Thetapapillomavirus 1*	AF502599.1	7304	*Psittacus erithacus* (African grey parrot)	PePV1	52.98	52.17
Fringilla coelebs papillomavirus 1	*Etapapillomavirus*	*Etapapillomavirus 1*	AY057109.1	7729	*Fringilla coelebs* (Common chaffinch)	FcPV1	50.30	49.76
Francolinus leucoscepus papillomavirus 1	*Dyoepsilonpapillomavirus*	*Dyoepsilonpapillomavirus 1*	EU188799.1	7498	*Pternistis leucoscepus* (Yellow-necked francolin)	FlPV1	50.78	50.46
Chelonia mydas papillomavirus 1	*Dyozetapapillomavirus*	*Dyozetapapillomavirus 1*	EU493091.1	6953	*Chelonia mydas* (Green sea turtle)	CmPV1	46.02	45.34
Caretta caretta papillomavirus 1	*Dyozetapapillomavirus*	*Dyozetapapillomavirus 1*	EU493092.1	7020	*Caretta caretta* (Loggerhead sea turtle)	CcPV1	46.22	45.43
Pygoscelis adeliae papillomavirus 1	*Treisepsilonpapillomavirus*	*Treisepsilonpapillomavirus 1*	KJ173785.1	7637	*Pygoscelis adeliae* (Adélie penguin)	PaPV1	51.71	51.47
Fulmarus glacialis papillomavirus 1	*Treiszetapapillomavirus*	*Treiszetapapillomavirus 1*	KJ452243.1	8132	*Fulmarus glacialis* (Northern fulmar)	FgPV1	45.32	44.19
Pygoscelis adeliae papillomavirus 2	*Treisepsilonpapillomavirus*	*Treisepsilonpapillomavirus 1*	MF168943.1	7654	*Pygoscelis adeliae* (Adélie penguin)	PaPV2	51.41	51.74
Serinus canaria papillomavirus 1	*Etapapillomavirus*	*unclassified Etapapillomavirus*	MF564196.1	8071	*Serinus canaria* (Atlantic canary)	ScPV1	50.35	50.13
Fratercula arctica papillomavirus 1	*unclassified*	*unclassified*	MK620302.1	7703	*Fratercula arctica* (Atlantic puffin)	FarcPV1	54.50	54.17
Duck papillomavirus 3	*unclassified*	*unclassified*	MK620303.1	7887	*Anas platyrhynchos* (Mallard)	AplaPV1	54.30	53.89
Larus smithsonianus papillomavirus 1	*unclassified*	*unclassified*	MK620304.1	7699	*Larus smithsonianus* (American herring gull)	LsmiPV1	54.30	54.04
Rissa tridactyla papillomavirus 2	*unclassified*	*unclassified*	MK620305.1	7763	*Rissa tridactyla* (Black-legged Kittiwake)	RtriPV1	54.57	54.20
Anas platyrhynchos papillomavirus 2	*unclassified*	*unclassified*	MW888448.1	8350	*Anas platyrhynchos* (Mallard or wild duck)	AplaPV2	45.28	45.79
Anas platyrhynchos papillomavirus 3	*unclassified*	*unclassified*	PP057987.1	7839	*Anas platyrhynchos* (Mallard or wild duck)	AplaPV3	56.66	56.39

**Table 2:** Detected PVs’ genome annotations and comparative analysis of ORF.

Gene Synteny	Genome Coordinates	nt Size	AA Size	Best Blast Hits (GenBank Accession Number)	Product	Similarity (%)	Note
Todiramphus sanctus papillomavirus 1 (TsPV1, GenBank accession no. PP922140.1)							
ORF01	318–629	312	103	Duck papillomavirus 3 (QBR99466.1)	E6	44.44	
ORF02	693–2801	2109	702	Duck papillomavirus 3 (QBR99468.1)	E1	64.84	
ORF03	823–1440	618	205	Serinus canaria papillomavirus 1 (YP_009551917.1)	E9	29.85	
ORF04	2722–3918	1197	398	Gull papillomavirus 1 (QBR99477.1)	E2	38.65	
ORF07	3939–5570	1632	543	Duck papillomavirus (ANN29878.1)	L2	45.36	
ORF08	5558–7108	1551	516	Duck papillomavirus 3 (QBR99472.1)	L1	65.05	
ORF06	7110–7556	447	148				hypothetical gene
Cacatua sanguinea papillomavirus 1 (CsPV1, GenBank accession no. PP922141.1)							
ORF01	318–629	312	103	Duck papillomavirus 3 (QBR99466.1)	E6	44.44	
ORF02	717–2825	2109	702	Duck papillomavirus 3 (QBR99468.1)	E1	64.84	
ORF03	808–1464	657	218	Serinus canaria papillomavirus 1 (YP_009551917.1)	E9	29.85	
ORF04	2746–3942	1197	398	Gull papillomavirus 1 (QBR99477.1)	E2	38.90	
ORF05	3950–5554	1605	534	Duck papillomavirus (ANN29878.1)	L2	44.90	
ORF06	5542–7092	1551	516	Duck papillomavirus 3 (QBR99472.1)	L1	65.05	
ORF07	7136–7516	381	126				hypothetical gene

**Table 3:** Average pairwise nucleotide identities of L1 gene among APVs.

		1	2	3	4	5	6	7	8	9	10	11	12	13	14	15	16	17
1	TsPV1-PP922140																	
2	CsPV1-PP922141	100																
3	AplaPV1-MK620303	63.14	63.14															
4	AplaPV2-MW888448	49.89	49.89	50.17														
5	AplaPV3-PP057987	64.24	64.24	72.73	51.68													
6	FlPV1-EU188799	55	55	61.75	49.10	61.84												
7	PePV1-AF502599	59.65	59.65	60.71	47.70	61.65	59.66											
8	RtriPV1-MK620305	60.13	60.13	60.42	48.55	59.75	57.16	58.16										
9	LsmiPV1-MK620304	57.81	57.81	60.32	49.23	59.97	57.49	57.12	68.38									
10	PaPV2-MF168943	56.19	56.19	58.70	49.44	58.23	55.47	56.91	59.06	60.51								
11	FarcPV1-MK620302	58.94	58.94	60.71	49.40	60.53	59.59	58.49	70.04	69.45	59.87							
12	PaPV1-KJ173785	56.74	56.74	60.89	51.35	59.02	59.04	57.20	59.53	59.41	65.15	62.60						
13	FcPV1-AY057109	59.72	59.72	59.95	50.35	59.20	55.33	56.57	55.44	54.84	54.48	57.07	54.38					
14	ScPV1-MF564196	60.08	60.08	59.37	49.41	60.01	54.62	56.84	56.57	55.08	54.34	56.65	54.64	64.86				
15	CmPV1-EU493091	49.83	49.83	51.77	47.95	50.71	48.60	50.96	48.44	47.88	50.24	50.07	50.07	47.81	49.62			
16	CcPV1-EU493092	51.72	51.72	52.92	48.69	52.95	50.27	49.83	50.85	48.59	50.83	51.68	50.31	49.21	51.58	70.91		
17	FgPV1-KJ452243	49.86	49.86	50.21	63.18	50.70	49.31	50.25	49.68	48.77	50.63	48.87	49.25	48.63	50.49	46.79	47.67	

## Data Availability

Nucleotide sequences and associated data for the TsPV1 and CsPV1 genomes are available in the DDBJ/EMBL/GenBank databases under accession numbers PP922140–PP922141. The raw sequencing data from this study have been deposited in the NCBI Sequence Read Achieve (SRA) under the accession numbers SRR31823170–SRR31823171 and the BioProject accession number: PRJNA1202390 (BioSample accession numbers: SAMN45957023–SAMN45957024).

## Supplementary Materials

The following supporting information can be downloaded at: https://www.mdpi.com/article/10.3390/pathogens14060514/s1, Table S1: Sampling details used in this study.
