# Supplementary material for: Discovery and Genomic Characterisation of Novel Papillomaviruses in Australian Wild Birds
Source: Pathogens. 2025 May 22;14(6):514. doi: 10.3390/pathogens14060514 (PMC12196351; doi:10.3390/pathogens14060514)
Supplement: Supplementary file 1 [file pathogens-14-00514-s001.zip › pathogens-3644240-supplementary.pdf]

**Supplementary File**

# Discovery and genomic characterisation of novel papillomaviruses in Australian wild birds

**Subir Sarker[a*], Vasilli Kasimov[b], Md. Mizanur Rahaman[a], Babu Kanti Nath[c] and Martina Jelocnik[d,e]**

[a] Biomedical Sciences & Molecular Biology, College of Medicine and Dentistry, James Cook University, Townsville, QLD 4811, Australia; subir.sarker@jcu.edu.au (S.S.)
[b] Institute for Biomedicine and Glycomics, Griffith University, Southport, QLD 4215, Australia; v.kasimov@griffith.edu.au (V.K.)
[c] Biosecurity Research Program and Training Centre, Gulbali Institute, Charles Sturt University, Wagga Wagga, NSW, Australia.
[d] School of Science, Technology and Engineering, University of the Sunshine Coast, Sippy Downs 4557, Australia; mjelocni@usc.edu.au (M.J.)
[e] Centre for Bioinnovation, University of the Sunshine Coast, Sippy Downs 4557, Australia.

* Correspondence: subir.sarker@jcu.edu.au

**Supplementary Table S1.** Sampling details used in this study

| **Type** | **Number** | **Identity** | **Sample_site** | **Date** | **Scientific name** | **Family** | **Location** | **Admission cause (adjusted)** | **BFDV_qPCR** | **PsAHV1_qPCR** | ***Avipoxvirus*_qPCR** | **CoAHV1_qPCR** | ***Chlamydiaceae*_qPCR** |
|---|---|---|---|---|---|---|---|---|---|---|---|---|---|
| Pigeon | 1259465 | Crested Pigeon | Liver | 14/2/2021 | Ocyphaps lophotes | Columbidae | Rochedale South | Clinical disease (emaciated) | + | + | + | + | - |
| Pigeon | 104111 | Crested Pigeon | Liver | 5/3/2021 | Ocyphaps lophotes | Columbidae | Marcoola | Trauma (unsp.) | + | + | + | + | - |
| Pigeon | 104925 | Crested Pigeon | Liver | 19/3/2021 | Ocyphaps lophotes | Columbidae | Currimundi | Trauma (unsp.) | + | + | + | + | - |
| Pigeon | 110703E | Crested Pigeon | Eye/Choana | 30/10/2021 | Ocyphaps lophotes | Columbidae | Caboolture | Trauma (fracture) | + | + | + | + | - |
| Kingfisher | 99207EC | Sacred Kingfisher | Eye/Choana/Cloaca | 9/10/2020 | Todiramphus sanctus | Alcedinidae | Peachester | Trauma (other) | - | + | + | + | - |
| Kingfisher | 110604E | Sacred Kingfisher | Eye/Choana | 29/10/2021 | Todiramphus sanctus | Alcedinidae | Caboolture | Trauma (fracture) | + | + | + | + | - |
| **Kingfisher** | **99207EC** | **Sacred Kingfisher** | **Eye/Choana** | **9/10/2020** | **Todiramphus sanctus** | **Alcedinidae** | **Peachester** | **-** | **-** | **-** | **-** | **-** | **-** |
| Parrot | 100234E | Little Corella | Eye/Choana | 1/11/2020 | Cacatua sanguinea | Cacatuidae | Buderim | Other | - | + | + | + | + |
| **Parrot** | **99304E** | **Little Corella** | **Eye** | **10/11/2020** | **Cacatua sanguinea** | **Cacatuidae** | **Burpengary** | **-** | **-** | **-** | **-** | **+** | **+** |
| Parrot | 99843E | Long-billed Corella | Eye/Choana | 22/10/2020 | Cacatua tenuirostris | Cacatuidae | Caboolture | Clinical disease (BFD) | + | + | + | + | - |
| Raptor | 110921E | Barn Owl | Eye/Choana | 4/11/2021 | Tyto alba | Tytonidae | NA | Clinical disease (emaciated) | + | - | + | + | - |
